# Design and Application of Antifouling Bio-Coatings

**DOI:** 10.3390/polym17060793

**Published:** 2025-03-17

**Authors:** Jinglin Wang, Ling Li, Yage Wu, Yongchun Liu

**Affiliations:** Key Laboratory of Applied Surface and Colloid Chemistry, Ministry of Education, School of Chemistry and Chemical Engineering, Shaanxi Normal University, Xi’an 710119, China; jljl@snnu.edu.cn (J.W.); lilingling@snnu.edu.cn (L.L.); wuyage@snnu.edu.cn (Y.W.)

**Keywords:** bio-coating, antifouling, biopolymer

## Abstract

Antifouling coatings stand out as one of the highly efficient ways to mitigate surface contamination. Traditional antifouling coatings have a major drawback: they rely on highly toxic and environmentally hazardous compounds. These substances not only lead to ecological harm but also disrupt the natural equilibrium of ecosystems. Consequently, in recent years, eco-friendly antifouling bio-coatings have emerged. This review focuses on the mechanisms and processes underlying contaminant adhesion, laying a solid foundation for grasping the principles of antifouling coating design. It further elaborates on the general strategies for developing bio-based antifouling solutions, highlighting their potential across a wide array of applications. Finally, this review carefully analyzes the current challenges confronted by antifouling bio-coatings and puts forward future development directions. Through a comprehensive overview, we aim to expand the influence of bio-based antifouling technologies, promote the further application of bio-based antifouling coatings in marine antifouling and medical antifouling fields, and provide examples for the establishment of environmental protection policies.

## 1. Introduction

**Figure 1:**
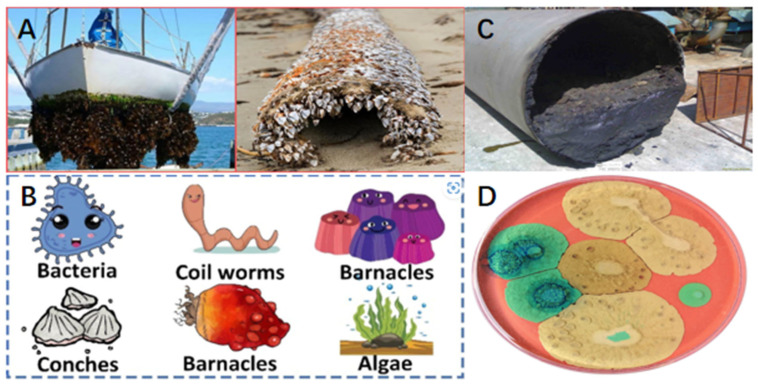
(**A**) Bioaccumulation on the surface of a ship hull. (**B**) Biological fouling on the surface of a ship, including bacteria, barnacles, mussels, sea snails, algae, etc. (**C**) Impurities adhering to the inner wall of a pipeline. (**D**) Medical equipment surfaces containing adherent microbial communities.

Interface contamination, a major problem in many fields, is the movement, change, and accumulation of pollutants at the contact points between different materials, such as solid–liquid, gas–liquid, and solid–gas interfaces. This happens because pollutants are drawn to the interface by residual forces. Interface contamination causes significant economic losses and increases safety risks, especially in marine transportation, pipelines, and healthcare applications. In transportation, microorganisms and pollutants gradually colonize ship surfaces (Figure 1A,B), disrupting fluid dynamics, increasing fuel consumption, and raising maintenance and repair costs [1]. Similarly, in pipeline systems, scaling and oil adhesion reduce transport efficiency, increase energy consumption, and pose safety hazards (Figure 1C) [2,3]. In the medical field [4,5,6], the adhesion of cells and bacteria on implants can cause infections (Figure 1D) [7,8,9], while contamination of blood transfusion tubes by fibrinogen leads to thrombus formation, compromising transfusion efficiency and endangering patients’ lives [10,11,12,13].

The issue of antifouling emerged earliest on ships [14]. Bio-fouling on ships has been a persistent problem for sailors, which has driven the emergence of antifouling technologies. The ancient Phoenicians were the earliest inventors of antifouling coatings, using lead plates to cover the hulls of ships. In modern times, the widespread use of copper- and tin-based antifouling coatings has caused certain damage to the marine environment. Antifouling coatings are developing towards being nontoxic and harmless [15].

In recent years, significant progress has been made in the development of antifouling bio-coatings, which are prepared from biopolymers such as polysaccharides or proteins through physical, chemical, and biological methods. Compared with traditional antifouling coatings, antifouling bio-coatings offer five key advantages: (1) Bio-coatings are composed of biomass, making them biodegradable and nontoxic, thus avoiding long-term environmental pollution [3]. (2) They can integrate multiple functions, such as antibacterial and antiviral properties, broadening their application fields [16]. (3) Their nontoxic nature allows for use in human-related fields, such as medical devices [17]. (4) Their raw materials are renewable, aligning with sustainable development and environmental regulations [18]. (5) Their development has driven research into new antifouling mechanisms, such as micro-nano interface mechanisms and interface self-renewal mechanisms.

The development of bio-based antifouling materials aligns with global sustainability goals and industrial needs. This review aims to elucidate the processes and mechanisms underlying interface contamination while introducing novel bio-based antifouling surfaces. It highlights the latest advancements in bio-based antifouling materials and delineates their emerging application domains. Unlike traditional antifouling coatings and their application directions in marine environments, we have introduced the potential application value of bio-based antifouling coatings in more fields. By doing so, this work seeks to underscore the importance of continued innovation in this field and its broader implications for environmental and societal well-being.

## 2. Mechanisms of Contaminant Interface Adhesion

### 2.1. The Interaction Between Contaminant and Interface

A deep understanding of the interaction between pollutants and interface helps in the targeted development of antifouling coatings. The adhesion of pollutants to an interface depends on the chemical, physical, and mechanical interactions between the interface and the pollutants.

**Figure 2:**
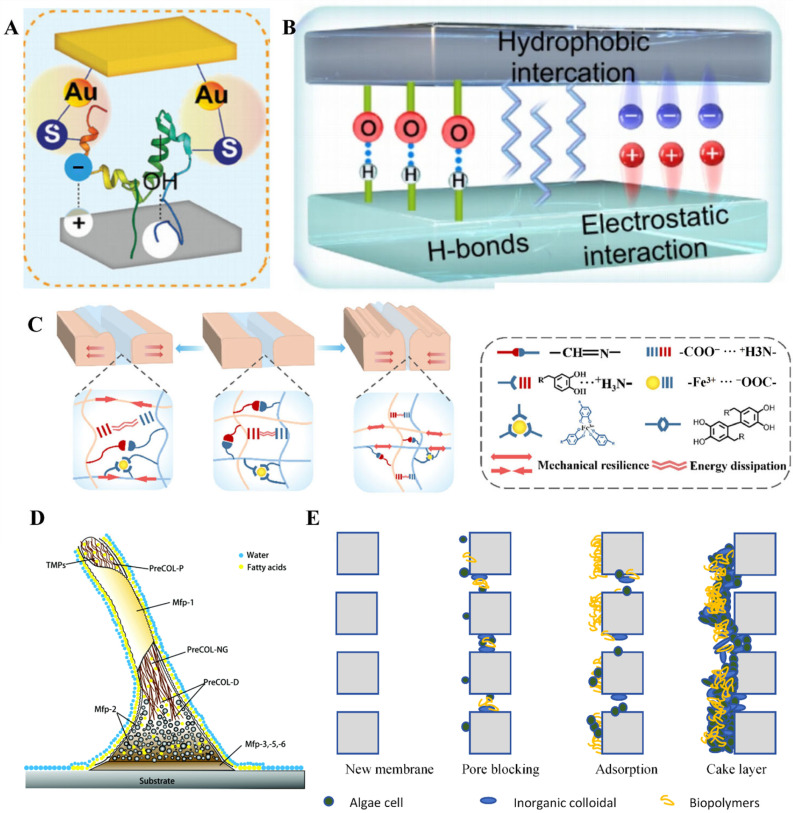
(**A**) The gold atom forms a gold–sulfur bond with the sulfhydryl group of the protein. Copyright 2024, Wiley-VCH GmbH. (**B**) Nonbonding interactions between proteins and interfaces, including hydrophobic, van der Waals forces, hydrogen bonding, electrostatic forces. (**C**) Multiple interactions of tissue adhesion. Copyright 2023, Elsevier. (**D**) Drainage of long-chain fatty acids during mussel adhesion. Copyright 2018, Royal Society of Chemistry. (**E**) Blockage and contamination of permeable membranes caused by interlocking.

Chemical factors include covalent bonds, ionic bonds, and coordination bonds. For example, the adhesion process of proteins on a gold surface [19]. Proteins are organic macromolecules composed of various amino acids. Cysteine, which is rich in thiol groups, can stably bind to the gold surface through gold–thiol bonds, causing contamination of the gold interface (Figure 2A). Mussels achieve robust underwater adhesion through the coordination of dopamine with iron ions and cross-linking protein networks [20]. The amino groups on the surface of microorganisms can undergo condensation reactions with the aldehyde groups at the interface, forming Schiff bases and causing interface contamination (Figure 2C). Fatty acid molecules interact with amino or hydroxyl groups on the surface, forming ester or amide bonds, which securely attach lipid molecules to the surface.

Physical adsorption includes hydrogen bonds, van der Waals forces, hydrophobic interactions [21], as well as surface rearrangement [22]. Lipids and other organic compounds can remove interfacial water through hydrophobic interactions and then bind to the interface through van der Waals forces, forming a layer of lipid films that are difficult to remove. They may further adsorb other pollutants, causing interface contamination [23,24]. Long-chain fatty acids and linear polyol lipids are the main components of lipid adhesion. Studies have shown that long-chain fatty acids play an important role in the underwater adhesion process of mussels and barnacles. They are first secreted to replace interfacial water, followed by synergistic action with proteins to produce adhesive effects (Figure 2D) [25,26,27,28,29]. Proteins interact with surfaces through multiple mechanisms: hydrophobic amino acids first expel water, followed by hydrophilic amino acids forming hydrogen bonds and van der Waals interactions. (Figure 2B).

Mechanical interactions mainly include interlocking [21,30]. The contamination of separation membrane surfaces occurs through mechanical interactions between aqueous particles and colloidal substances with the membrane during filtration. These interactions induce adsorption and deposition onto or within the membrane pores, culminating in membrane blockage [31]. Taking polyvinylidene fluoride (PVDF) membrane as an example (Figure 2E), during the wastewater treatment process, its surface is easily affected by macromolecular proteins and high concentrations of inorganic ions in the wastewater solution, leading to blockage of PVDF membrane pores, surface contamination, and a significant decrease in permeate flux [32].

### 2.2. The Adhesion Process of Interface Contaminant

**Figure 3:**
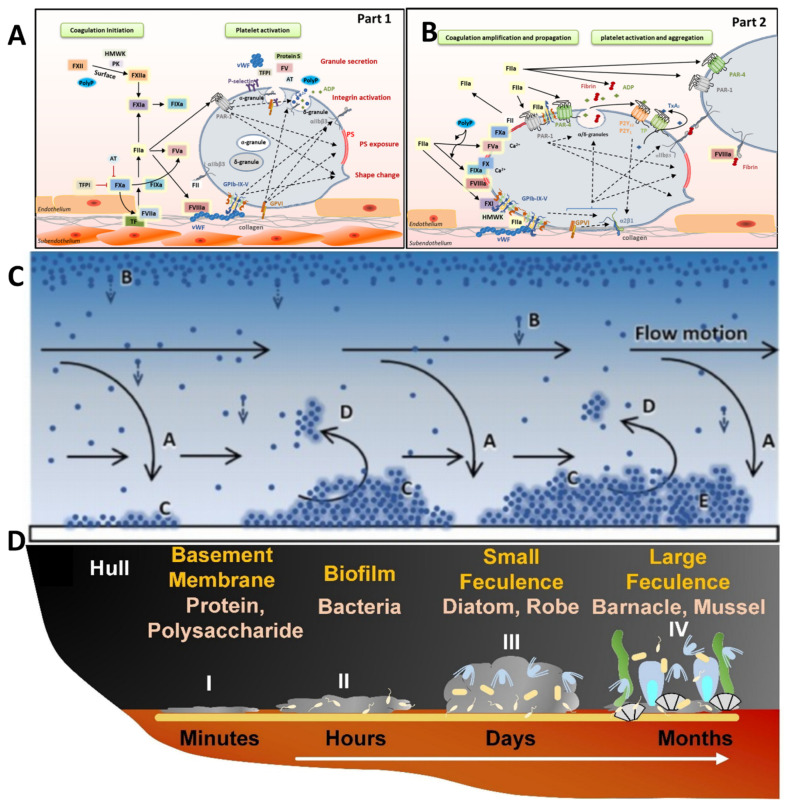
(**A**,**B**) Cell adhesion process. Copyright 2021, Elsevier. (**C**) Industrial pipeline fouling process. (Bio)fouling development. Mature fouling (E) results from the balance between deposition forces (convective (A) and diffusion (B) transport, physical, chemical and biological attachment (C)) and removal forces (shear effects, D). Copyright 2021, Elsevier. (**D**) Adhesion processes of marine organisms. Copyright 2024, Elsevier.

The adhesion process of contaminants has typical spatial and temporal characteristics (Figure 3D). It always involves the initial coating of the surface by organic macromolecules, followed by the adhesion of microorganisms or cells. Understanding these dynamic interaction behaviors is important for developing efficient antifouling strategies to mitigate contamination.

In most instances of marine biological contamination, the interface is initially coated by macromolecules, such as proteins and polysaccharides. This coating provides a suitable microenvironment for bacteria growth [33]. These microorganisms secrete a large amount of metabolic products on the interface, altering the microscopic morphology of the interface further. The new surface composed of metabolic products provides abundant nutrients and adhesion sites for other marine organisms, promoting interface contamination [34]. The adhesion process of mussels has been extensively studied. Mussels first use their foot’s tactile sensors to find a suitable substrate, then secrete adhesive proteins rich in high concentrations of dihydroxyphenylalanine (DOPA), firmly adhering to the hull [35,36]. The adhesion of algae occurs through a two-step process: first, algal cells synthesize and release polysaccharides and glycoprotein adhesives, such as alginate, which form a flexible network [37,38]. Second, these adhesives undergo covalent cross-linking and solidify, catalyzed by substances like peroxidase, ultimately enabling algae to firmly adhere to the substrate. For another marine fouling organism, the barnacle, its adhesion process also involves multiple steps [39]. Initially, barnacle larvae attach to surfaces, forming temporary adhesion. Subsequently, gland cells within the barnacles secrete a series of adhesive proteins, which are transported through ducts to the interface. Following the exchange of water layers, these proteins combine and solidify with the surface, establishing strong underwater adhesion.

In the medical field, unnecessary cell adhesion and thrombus formation on the surface of medical devices also exhibit typical spatial and temporal characteristics. When cells contact the interface, they secrete an extracellular matrix composed of proteoglycans and glycosaminoglycans, which adhere to the device’s surface. This is followed by adhesion through cell adhesion molecules such as cadherins, selectins, integrins, and hyaluronan adhesin [40]. Thrombus formation on medical devices is triggered by the activation of prothrombin by calcium ions, converting fibrinogen into insoluble fibrin polymers that interweave into a network, increasing blood viscosity and promoting blood cell adhesion [41,42]. Platelet adhesion is a key event in thrombus formation (Figure 3A,B), involving multiple signaling molecules such as glycoprotein IB-IX-V and P-selectin. The von Willebrand factor (vWF) serves as a bridge for platelet adhesion to tissues, slowing platelet flow and triggering intracellular signal transduction, leading to firm adhesion at the tissue interface [43,44].

In pipeline systems, scaling and oil adhesion also have spatial and temporal characteristics. [45] Initially, the continuous deposition of calcium and magnesium ions in the solution leads to the formation of scale. This scale acts as a conditioning layer, altering the inner wall of the pipe and making it rough. Subsequently, pollutants such as oils deposit at the scaled areas, forming particulate matter, and finally, these particulates continuously grow in size. Ultimately, this results in the blockage of the pipe (Figure 3C) [46].

## 3. Antifouling Mechanisms

Shielding adhesive forces and interrupting the adhesion process are key principles guiding antifouling coatings research. Natural-surface-inspired antifouling strategies have made significant progress, providing valuable insights for antifouling coatings. Current antifouling strategies mainly rely on the following four mechanisms: (a) adjusting surface energy, [47]; (b) creating superhydrophilic or superhydrophobic surfaces, [48]; (c) introducing microstructures on the surface, [49]; and (d) constructing superslippery surfaces. [50] Ultimately, these mechanisms aim to control how contaminants adhere to or detach from material surfaces.

### 3.1. Surface Free Energy

**Figure 4:**
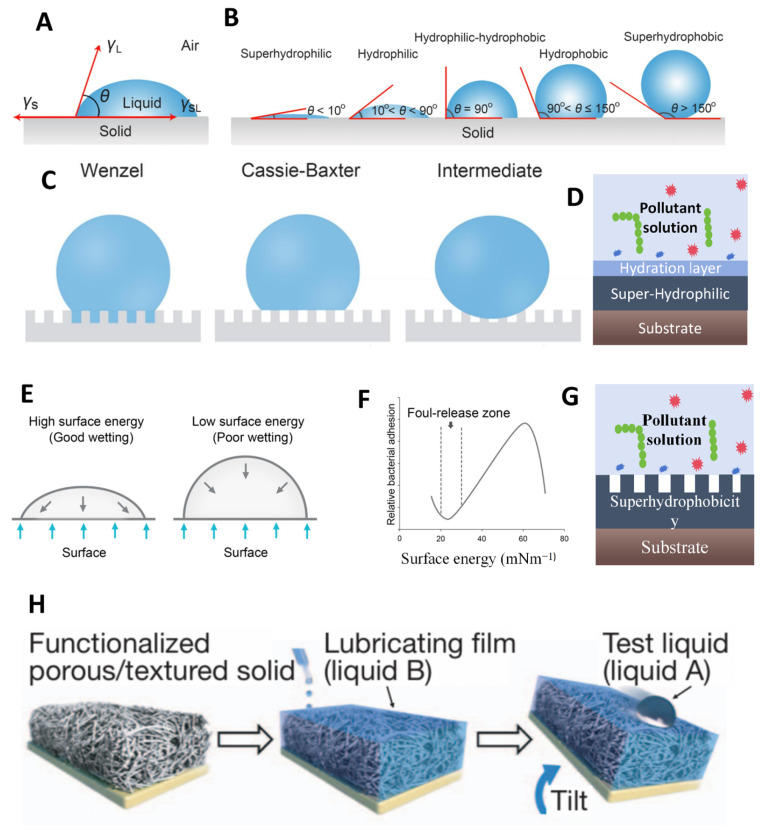
(**A**) Schematic diagram of the definition of contact angle. (**B**) Different states of solid–liquid contact angles. (**C**) Wenzel state, Cassie–Baxter state, Intermediate state. (**D**) Superhydrophilic antifouling mechanism. (**E**) Wetting states with interfaces of different surface energies. (**F**) Classical “Baier curve”. Copyright 2022, Elsevier. (**G**) Superhydrophobic antifouling mechanism. (**H**) Schematic diagram of the strategy for injecting lubricating liquid into the interface. Copyright 2021, Elsevier.

Surface energy, defined as the reversible work required to increase the surface area under isothermal, isobaric, and constant composition conditions, is a fundamental parameter for characterizing interfacial properties (Figure 4E). The relationship between surface free energy and adhesion phenomena has been extensively studied. Baier’s seminal work establishes a well-defined correlation between surface energy and contaminant adhesion, as illustrated by the famous Baier curve [51] (Figure 4F). This curve demonstrates that the best antifouling performance occurs within a specific surface energy range of 22–24 mJ/m^2^ [52,53]. In an aqueous system, water must rewet the system when proteins and cells are removed. For solids with a surface energy of ∼22 mN/m, the thermodynamic “cost” for water to re-wet the surface is minimized. From a wettability standpoint, interfaces with high surface energy typically display improved liquid wettability, and vice versa. Thermodynamically, contamination adhesion on low-surface-energy surfaces will cause an energy shift towards higher states, which is energetically unfavorable. This natural thermodynamic resistance contributes to the superior antifouling performance of low-surface-energy materials. In the industrial field, low-surface-energy antifouling technologies have reached a relatively mature stage. Materials such as polydimethylsiloxane (PDMS) have been demonstrated to exhibit effective antifouling capabilities through chemical modifications, which create stable low-surface-energy interfaces, thereby resisting microbial colonization and particulate adhesion [54]. However, it is important to note that the antiadhesion properties of low-surface-energy interfaces are not absolute. Microbial adhesion represents a complex, dynamic process influenced by multiple factors, necessitating comprehensive evaluation and integrated approaches for effective implementation.

### 3.2. Surface Wettability

Wettability refers to the ability and tendency of the water to spread on a solid surface [55] typically reflected by the contact angle (Figure 4A). According to Young’s equation, interfaces with a macroscopic stable contact angle of less than 5° are defined as superhydrophilic interfaces. When the contact angle is greater than 90° but less than 150°, it is defined as a hydrophobic interface; when the contact angle is greater than 150°, it is defined as a superhydrophobic interface (Figure 4B) [56,57]. Although wettability and surface energy can be related through Young’s equation, wettability is more concerned with the behavior of water at the interface. Therefore, the influence of wettability on antifouling will be discussed separately here. Currently, antifouling mechanisms based on interfacial wettability can be divided into two directions: superhydrophilicity and superhydrophobicity.

Superhydrophobic surfaces exhibit remarkable water-repellent properties, causing liquids to form near-spherical droplets upon contact. This surface facilitates the strong cohesive forces between water molecules, which dominate the adhesive forces between water and the solid, driving the water into adopting a spherical configuration that minimizes the liquid–solid contact area [58]. The minimized contact area significantly reduces water adhesion to the surface, consequently decreasing the attachment and accumulation of surface contaminants (Figure 4G) [59]. Furthermore, as the water maintains a near-spherical morphology on the superhydrophobic surface, its mobility across the surface is increased. This characteristic behavior is referred to as the “roll-off effect”. The droplet moves across the superhydrophobic surface and effectively entraps and removes contaminants, thus demonstrating a self-cleaning capability. A representative example of this strategy is demonstrated by the superhydrophobic coating fabricated through spray deposition of polyvinylidene fluoride-hexafluoropropylene copolymer (PVDF-HFP). This coating significantly minimizes the interfacial contact area between contaminants and the substrate surface. The resultant surface properties not only reduce contaminant adhesion but also facilitate the removal of deposited particles through water droplet movement, thereby achieving efficient antifouling performance [60].

Superhydrophilic surfaces promote the formation of a dense and well-ordered hydration layer within a highly organized tetrahedral network structure through strong hydrogen (Figure 4D) [54]. This stable hydration layer serves dual functions: it completely covers the interface surface while simultaneously establishing an effective physical barrier against external contaminants. As a result, the direct contact between contaminants and the underlying surface is prevented, thereby significantly inhibiting contaminant adhesion and accumulation [61]. Furthermore, this hydration layer demonstrates dynamic stability through continuous water molecule exchange, which facilitates rapid self-renewal upon contaminant exposure, thereby enhancing the interface’s antifouling performance. A representative application of this mechanism is found in polyethylene glycol-based surface modification technology. When polyethylene glycol chains are immobilized on the interface, their abundant ether oxygen atoms facilitate the formation of an exceptionally stable hydration layer, which effectively blocks molecular interactions between contaminants and the underlying surface [62].

### 3.3. Surface Microstructure

Microstructures are one of nature’s key ways of reducing fouling. Research has shown that the effectiveness of microstructures in preventing fouling depends on factors like size, shape, spacing, and height, which can increase or decrease adhesion. Some microstructure designs have been found to lower the attachment strength of certain organisms. Nature provides numerous exemplary models of microstructure-based antifouling strategies, particularly in aquatic environments [63]. The antifouling mechanism of engineered surface microstructures primarily involves the strategic design of micro- and nanoscale geometric features to inhibit contaminant adhesion [64].

The antifouling effect of surface microstructures usually comes from several combined actions. By designing micro- and nanoscale bumps and indentations, these structures reduce the contact area between contaminants and the surface. A typical example is the lotus leaf-inspired design, where small bumps create a surface that limits contact with water droplets and contaminants to small points, reducing the chances of fouling.

The microstructured surface can also significantly enhance the hydrophobicity or hydrophilicity, thereby creating an unfavorable environment for contaminant adhesion. The influence of surface microstructures on wettability can be quantitatively described by the Cassie–Baxter equation, which models the wetting behavior of porous surfaces (Figure 4C) [51]. This theoretical framework treats liquid contact on rough surfaces as a composite interaction, where incomplete wetting leaves air pockets trapped within the porous architecture, forming a solid–liquid–gas triphase interface that ultimately reduces overall surface wettability. This principle is extensively utilized in self-cleaning materials, particularly superhydrophobic surfaces, where rolling water droplets can effectively remove surface-bound particulates [65].

The physical barrier effect is another crucial antifouling mechanism for microstructured surfaces. Engineered microstructures can physically obstruct or redirect contaminant flow, preventing their deposition. This principle is exemplified by shark skin’s surface design, where precisely arranged microstructures manipulate fluid dynamics to minimize contaminant accumulation [66]. Additionally, surface microstructures can effectively inhibit microbial reproduction through mechanical or electrochemical interactions. Certain nanostructures, for instance, can disrupt bacterial cell wall integrity, thereby preventing biofilm formation. This mechanism demonstrates particular efficacy against biological fouling, offering a promising approach for marine and medical applications.

### 3.4. Surface Superslippery

Superslippery surfaces represent a significant advancement in antifouling technology [67], which primarily manifests in two distinct forms: solid-state and liquid-state superslippery systems [68]. The solid superslippery surface generally refers to layered materials with an incommensurate interface, including graphene [69], molybdenum disulfide (MoS_2_) [70], and hexagonal boron nitride (hBN) [71,72]. These defect-free nanoscale 2D materials display extremely low surface energy defects, which minimize available adsorption sites for contaminants, thus achieving effective antifouling properties. Liquid-infused superslippery surfaces, alternatively, are created by impregnating micro/nanostructured surfaces with lubricating molecules, forming a protective interfacial layer known as Slippery Liquid-Infused Porous Surfaces (SLIPS) (Figure 4H). This innovative approach draws inspiration from natural biological systems, particularly the lubricated trapping surfaces of pitcher plants. By engineering artificial surfaces that mimic these natural structures, researchers have developed effective antifouling solutions.

The super-lubrication-based antifouling coating functions through two primary pathways: atomic-level smoothness and layered interface design. The atomically smooth structure eliminates reactive sites that could interact with contaminants, substantially reducing interfacial bonding and mechanical interlocking. For instance, hexagonal boron nitride (hBN)-based antifouling materials demonstrate exceptional performance due to their atomically smooth surfaces and polar boron-nitrogen bonds, which facilitate the formation of a dense hydration layer that inhibits heterogeneous nucleation and crystal adhesion [73]. Furthermore, the infusion of ultra-slippery liquids into surface micro/nanostructures creates self-regenerating lubricating coatings that effectively resist contamination. This mechanism is exemplified by pitcher plants, which maintain a lubricated nanostructured surface within their trapping apparatus, preventing contaminant adhesion through continuous liquid renewal [74].

## 4. Strategies for Constructing Antifouling Bio-Coatings

Bio-based materials, made from renewable resources through biological, chemical, or physical processes, provide a sustainable alternative to traditional materials. These materials come from various natural sources, such as crops, forestry products, and plant and animal byproducts. Known for being eco-friendly, renewable, and biodegradable, bio-based materials have clear benefits for the environment. Antifouling coatings made from these materials not only offer effective surface protection but also support global environmental goals, providing a green solution that balances technology with ecological responsibility.

### 4.1. Superhydrophilic Interface

The fabrication of superhydrophilic interfaces applies surface wettability principles to achieve enhanced water affinity and functional performance [75]. These interfaces exhibit exceptional water affinity, leading to the formation of a unique hydration layer at the surface interface [76]. Unlike bulk water molecules, this interfacial water layer possesses a highly ordered tetrahedral coordination structure, which creates an effective barrier against contaminant adhesion [77]. Furthermore, the disruption of this structured hydration layer by contaminants is thermodynamically unfavorable, thereby endowing superhydrophilic interfaces with remarkable antifouling properties. The current strategies for constructing bio-based superhydrophilic coatings include polymer blending strategies, phase transition self-assembly strategies, chemical deposition strategies, and spin-coating strategies, among others. They have their own advantages in terms of the potential for large-scale production with high structural accuracy and are suitable for different application directions (Table 1).

#### 4.1.1. Polymer Blending Strategy

**Figure 5:**
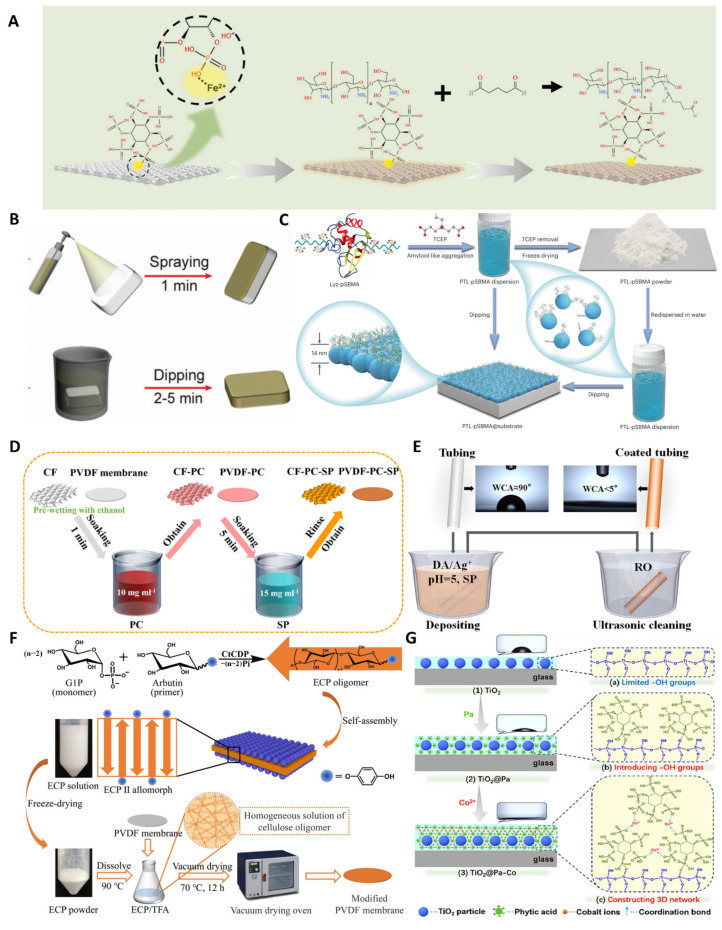
(**A**) Polymer blending strategy for preparing CS-SSM coatings. Copyright 2023, Elsevier. (**B**) PTL-PEG coatings prepared by phase transition assembly methods. (**C**) PTL-pSBMA coatings prepared by phase transition self-assembly methods. (**D**) Anthocyanin coatings prepared by chemical deposition strategies. Copyright 2022, Elsevier. (**E**) PDA/Ag coatings prepared by chemical deposition methods. Copyright 2022, Elsevier. (**F**) Cellulose-based superhydrophilic coatings prepared by spin-coating strategies. Copyright 2024, Elsevier. (**G**) TiO_2_-Pa coatings prepared by spray-coating and dip-coating strategies. Copyright 2020, Elsevier.

Building upon the fundamental principles of superhydrophilic coatings, Xue’s research team developed a polymer blending approach to fabricate chitosan-based hydrogel coatings on iron substrates (Figure 5A) [78]. The coating system comprises two key components: phytic acid and chitosan, which synergistically contribute to its functionality. Phytic acid serves a dual purpose by chelating with surface metal ions while simultaneously introducing abundant hydroxyl groups at the interface. This modified surface then facilitates the electrostatic immobilization of chitosan, which undergoes subsequent cross-linking and curing with glutaraldehyde to form the final CS-SSM coating. The superhydrophilic nature of this coating confers exceptional oil-repellent properties, making it particularly effective for water–oil separation applications. The fabrication strategy employs phytic acid to simultaneously modify the interface and generate abundant active sites, thereby establishing a reactive platform for subsequent chitosan modification. Furthermore, the formation of multiple chemical bonds between the coating components ensures robust adhesion to the substrate surface. Nevertheless, the coating’s substrate specificity remains a significant limitation that hinders its large-scale industrial implementation.

#### 4.1.2. Protein Phase Transition Self-Assembly Strategy

PEG and its derivatives are currently the most widely used antifouling materials. Yang et al. [79] adopted a phase transition self-assembly strategy to construct protein coatings (Figure 5B). Through chemical grafting, lysozyme was PEGylated (Lyso-PEG). Using TCEP to reduce the intramolecular disulfide bonds of lysozyme triggered the corresponding amyloid aggregation of Lyso-PEG. The rapid amyloid aggregation effectively coats the substrate surface, forming a stable protein-anchored polyethylene glycol (PEG) coating that establishes a robust hydration layer, thereby imparting exceptional antifouling properties. The interface modification materials prepared through protein phase transition exhibit excellent biocompatibility. Moreover, the inherent adhesive capability of the β-sheet structure enables strong binding to various interfaces, making it suitable for diverse surface modification requirements. The in situ phase transition approach significantly simplifies the modification process, offering considerable potential for large-scale industrial applications.

Zwitterionic materials exhibit strong hydration through ionic solvation, endowing the material with excellent protein-resistant properties. Yang et al. [80] prepared zwitterionic protein coatings through a phase transition self-assembly strategy (Figure 5C). First, poly (sulfobetaine methacrylate) (pSBMA) was synthesized, followed by the coupling of lysozyme and pSBMA to prepare protein-polymer conjugates (Lyz-pSBMA). After reducing the intramolecular disulfide bonds of Lyz-pSBMA with tris(2-carboxyethyl)phosphine (TCEP), corresponding amyloid aggregates of Lyz-pSBMA rapidly formed stable two-dimensional phase-transition Lyz-pSBMA nanofilms (PTL-pSBMA) on various substrates. The hydrophilic layer demonstrates exceptional surface wettability, resulting in remarkable underwater superoleophobic properties. When applied to textile substrates, these hydrophilic polymer coatings enable autonomous oil stain removal through water rinsing alone, eliminating the need for chemical detergents. This protein-based anti-oil-fouling technology offers multiple environmental benefits: it substantially reduces laundry detergent consumption, decreases associated carbon emissions, and provides a sustainable approach to achieving eco-friendly, low-carbon lifestyle practices.

#### 4.1.3. Chemical Deposition Strategy

The environmentally friendly plant polyphenol, proanthocyanidin (PC), has attracted widespread attention in the field of superhydrophilic materials due to its excellent adhesive properties. Zhang’s research group constructed a polyphenol coating through a chemical deposition strategy (Figure 5D) [81]. The fabrication process involves sequential immersion of the substrate in proanthocyanidin and sodium periodate solutions, resulting in a stable proanthocyanidin coating. This coating demonstrates exceptional oil–water separation efficiency, remarkable stability, and superior reusability. The simplicity of this method allows for substantial proanthocyanidin immobilization while maintaining exposed functional groups, thereby offering significant potential for additional surface functionalization.

Surface chemistry inspired by mussels has greatly facilitated the development of coatings. Wang’s research group developed a dopamine superhydrophilic coating (PDA/Ag) through a chemical deposition strategy (Figure 5E) [82]. This was achieved by immersing the substrate in a solution containing sodium periodate, hydrochloric acid, dopamine, and silver nitrate, which facilitated the rapid formation of a PDA/Ag coating. The resulting coating exhibits an exceptionally low water contact angle of 5° and demonstrates remarkable protein-repellent properties, coupled with significant antibacterial activity. While the preparation of polydopamine/silver ion composite coatings is relatively straightforward, potential biosafety concerns arise from silver ion release during coating degradation, which currently limits their applicability in biomedical fields.

#### 4.1.4. Spin-Coating and Dip-Coating Strategy

Cellulose, the most abundant natural raw material in the world, exhibits excellent biodegradability, biocompatibility, and stability. Wang’s research group prepared a cellulose-based superhydrophilic coating through an immersion-coating strategy (Figure 5F) [83]. The synthesis process involves enzymatic catalysis by Clostridium thermocellum (CtCDP), which facilitates the production of antimicrobial cellulose from β-D-glucose and glucose-1-phosphate (G1P) precursors. The synthesized cellulose is subsequently dispersed in an organic solvent and applied to the substrate surface through dip-coating. This innovative coating not only significantly enhances the substrate’s surface hydrophilicity but also confers both antifouling and antimicrobial functionalities. The preparation method demonstrates notable advantages in terms of simplicity and environmental compatibility, making it a promising candidate for scalable industrial applications.

Phytic acid is an organic phosphorus compound extracted from plant seeds. Yu et al. prepared a phytic acid/titanium dioxide composite coating (TiO_2_-Pa) through a spray dip-coating strategy (Figure 5G) [84]. The innovative approach involves incorporating nanotitanium dioxide particles into a three-dimensional, multicoordinated phytic acid network structure, which imparts exceptional superhydrophilic characteristics to the interface. The resulting coating exhibits an ultra-low water contact angle of 2° and demonstrates remarkable antifogging performance. This methodology provides a straightforward yet effective solution for developing bio-based superhydrophilic coatings with practical applications.

### 4.2. Superhydrophobic Surfaces

Superhydrophobic surfaces exhibit extreme liquid repellency due to their low wettability [85,86]. This property is attributed to the formation of an air layer between the solid surface and the solution, which creates high surface tension at the three-phase interface. Consequently, the penetration of microbes and other pollutants is significantly hindered, resulting in an effective antifouling mechanism [87,88]. To fabricate such superhydrophobic bio-coatings, several advanced strategies are employed, including chemical deposition, spray coating, sol–gel processes, and organic-inorganic hybridization.

#### 4.2.1. Spray Coating Strategy

**Figure 6:**
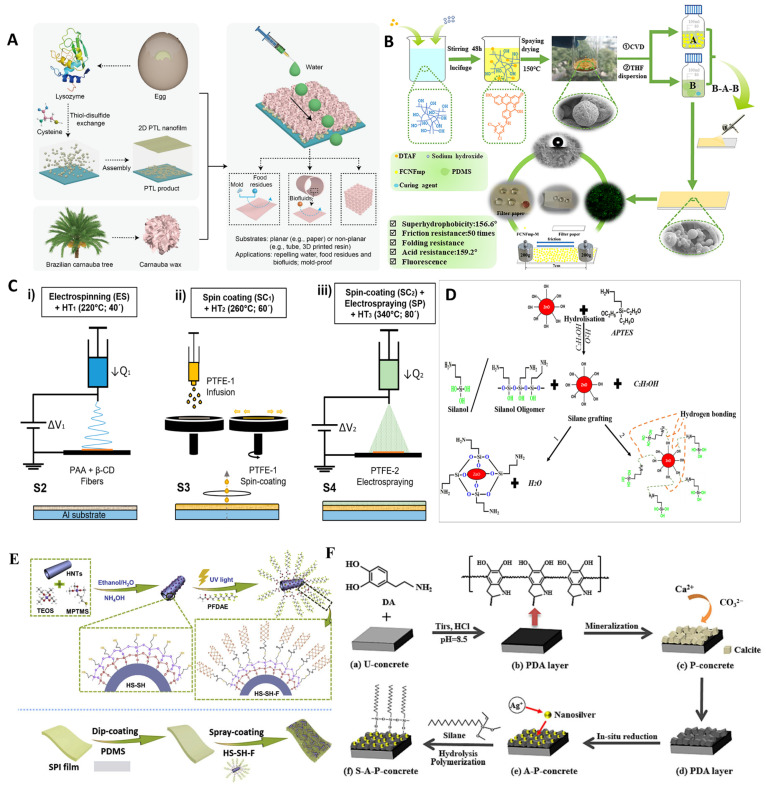
(**A**) Superhydrophobic coating of protein-palm wax composite prepared through spray coating strategy. Copyright 2021, Elsevier. (**B**) Cellulose/PDMS composite coating prepared through chemical deposition strategy. Copyright 2022, Elsevier. (**C**) Superhydrophobic coating of cyclodextrin prepared through electrospinning, spin coating, and electrospraying. (**D**) MZnO/HTPDMS-CPU was prepared by organic–inorganic hybrid strategy. Copyright 2022, Elsevier. (**E**) HS-SH@SPI superhydrophobic coating prepared by sol–gel method. Copyright 2019, Elsevier. (**F**) PDA/CaCO_3_ coating prepared through organic–inorganic hybridization strategy. Copyright 2023, Elsevier.

Yang’s group developed a new method to prepare bio-based superhydrophobic coatings using a spray coating strategy (Figure 6A) [89]. The process begins with the construction of a robust phase-transition lysozyme (PTL) coating on the substrate. Subsequently, a mixture of carnauba wax and hexane is prepared, heated, and spin-coated onto the PTL surface to form a protein-based superhydrophobic interface. The resulting composite coating, composed of proteins and carnauba wax, achieves a water contact angle of up to 150°, demonstrating excellent antiyogurt adhesion properties. This approach utilizes the inherent adhesiveness of protein coatings to create an intermediate adhesive layer, which enhances the binding of carnauba wax to the substrate while maintaining a straightforward fabrication process. The successful development of this superhydrophobic interface highlights its significant potential for large-scale applications.

Rafael et al. employed a spin-coating strategy to achieve a superhydrophobic coating on aluminum substrates through a three-step process involving electrospinning, spin-coating, and electrospraying (Figure 6C) [90]. The coating consists of polytetrafluoroethylene (PTFE), polyacrylic acid, and β-cyclodextrin. In this approach, the electrospun polyacrylic acid and β-cyclodextrin act as adhesive layers, effectively binding PTFE to the substrate surface. This results in a remarkable water contact angle of up to 170°, significantly improving the anticorrosion properties of the aluminum substrate. However, the intricate and multistep nature of the coating preparation process poses challenges for its large-scale implementation.

#### 4.2.2. Chemical Deposition Strategy

Cellulose is the most widely distributed and abundant polysaccharide in nature. Huang’s research group developed a cellulose-based superhydrophobic coating using a chemical deposition strategy (Figure 6B) [91]. In this method, MTMS and CNF were coupled by chemical vapor deposition, and then the coupling products and PDMS were sprayed on the substrate in turn. The assembly is then cured at 105 °C for 1 h to form the final superhydrophobic coating. The resulting coating exhibits remarkable mechanical stability, maintaining a water contact angle of 150° even after undergoing repeated abrasion tests. Furthermore, its exceptional acid resistance highlights its potential for outdoor applications, especially in environments where protection against acid rain corrosion is critical. The simple preparation method of the coating and its excellent mechanical stability provide potential for its industrial application.

#### 4.2.3. Sol–Gel Strategy

Li’s research group developed a new superhydrophobic interface using soy protein through a sol–gel strategy (Figure 6E) [92]. In this approach, silica was grown in situ on the surface of halloysite nanotubes (HNTs) via the sol–gel method, resulting in the formation of HS-SH particles. Subsequently, a composite solution of glycerol and soy protein was poured into a plastic petri dish to create a soy protein isolate (SPI) film. A layer of polydimethylsiloxane (PDMS) was then deposited onto the SPI film, followed by the spraying of dispersed HS-SH nanoparticles onto the surface to produce the HS-SH@SPI film. The resulting superhydrophobic film exhibited contact angles exceeding 150° for various liquids. This method capitalizes on the cost-effective self-film-forming properties of soy protein as a foundational material, while the incorporation of HNTs creates a nano/microstructured surface. The dual functionality of superhydrophobicity and superoleophobicity achieved by this approach enhances its practical value for diverse applications. However, the lengthy reaction of silica sol–gel makes it difficult for it to stand out among various superhydrophobic coatings and hinders its industrial application.

#### 4.2.4. Organic–Inorganic Hybridization Strategy

Dopamine is a biomolecule with excellent interface adhesion properties. Hou’s research group constructed a functional biomimetic mineralization hydrophobic coating using an organic-inorganic hybridization strategy (Figure 6F) [93]. In this approach, calcium carbonate was mineralized at the dopamine interface, while silver ions were simultaneously reduced. The process concluded with silane modification, resulting in the formation of a superhydrophobic coating. This coating demonstrates remarkable hydrophobicity, along with antibacterial and anti-icing properties, making it highly effective in protecting substrates from contamination in harsh marine environments. However, the complex and dynamic conditions of marine ecosystems may limit its potential for large-scale application.

Cashew phenol, a phenolic compound extracted from natural cashew nut shell oil, was utilized by Sahoo’s research team to develop a superhydrophobic coating through an organic-inorganic hybrid strategy (Figure 6D) [94]. This coating incorporates cashew phenol, silane coupling agents, and nanozinc oxide, exhibiting excellent superhydrophobicity with a water contact angle reaching 140°. Additionally, the coating demonstrates good mechanical properties, maintaining a high contact angle of 130° even after cyclic abrasion tests. While the organic-inorganic hybrid strategy addresses the issue of abrasion resistance in superhydrophobic coatings to some extent, its long-term performance still requires further evaluation.

### 4.3. Superlubric Interface

Superlubric interfaces represent an emerging and innovative direction in material development, inspired by the adaptive mechanisms of various organisms in nature. These interfaces function by introducing lubricating molecules between pollutants and solid surfaces, thereby achieving effective antifouling properties [95]. To construct such interfaces, a range of strategies have been employed, including chemical deposition, physical deposition, lubricating liquid injection, and other advanced techniques.

#### 4.3.1. Slippery Liquid-Infused Porous Surfaces Strategy

**Figure 7:**
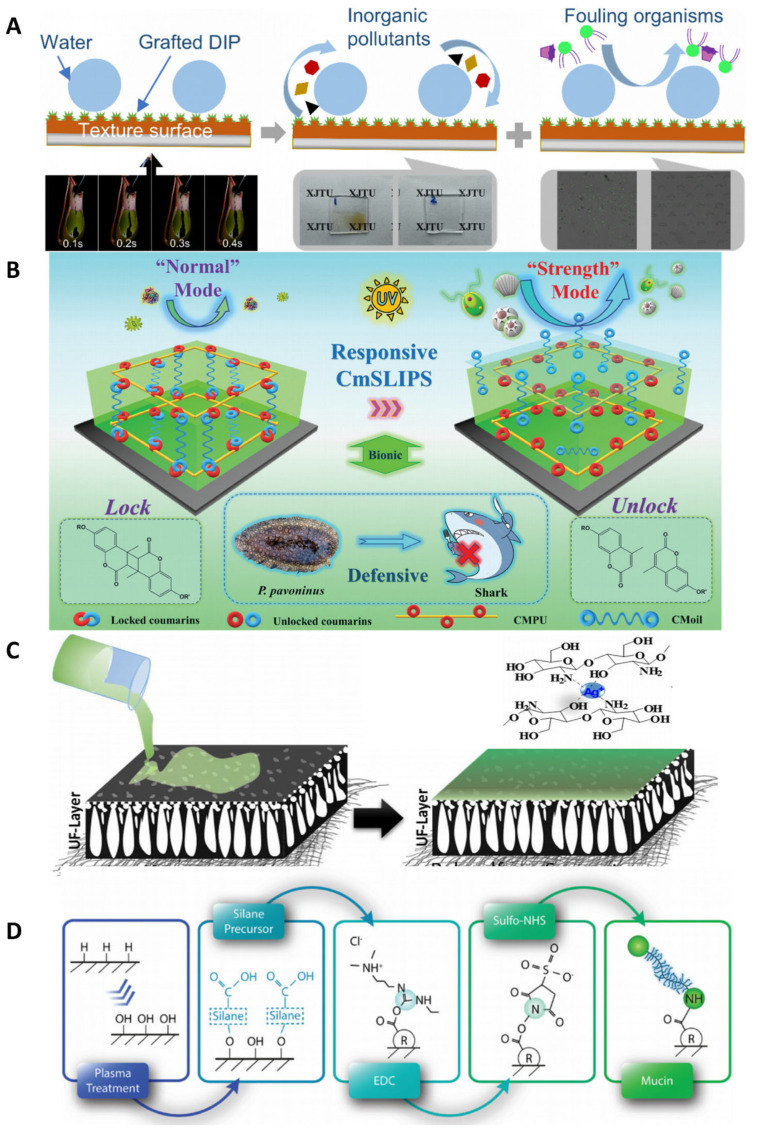
(**A**) Biomimetic pitcher plant antifouling interface prepared by lubrication liquid injection strategy. Copyright 2024, American Chemical Society. (**B**) Biomimetic shark skin antifouling interface prepared by lubrication liquid injection strategy. Copyright 2023, Wiley-VCH GmbH. (**C**) Chitosan silver nanoparticle coating prepared by physical deposition strategy. Copyright 2021, Elsevier. (**D**) Gastric mucin lubricating coating prepared by chemical deposition strategy.

The SLIPS strategy infuses lubricants into porous materials to form smooth, self-repairing surfaces that repel contaminants. Zhang’s research group has developed an innovative antifouling interface inspired by biomimetic pitcher plants, utilizing the Slippery Liquid-Infused Porous Surfaces (SLIPS) strategy (Figure 7A) [96]. Through the design of bionic textures carved into a poly(dimethylsiloxane) (PDMS) surface, they subsequently grafted a Dictyophora indusiata polysaccharide (DIP) modifier to fabricate the coating. The resulting smooth coating demonstrates exceptional antifouling performance against a variety of common contaminants, including Chlorella and coffee. This synergistic approach, which integrates environmentally friendly modifier grafting with a heterogeneous microstructure on the surface, opens up new possibilities for producing smooth coatings with remarkable protective properties.

Coumarin, a flavonoid compound isolated from soybean seeds, served as the foundation for Zhang’s group development of an intelligent marine antifouling interface using the SLIPS strategy (Figure 7B) [97]. By leveraging the reversible photodimerization properties of coumarin, they engineered reversible chemical bonds between the substrate and the lubricant. This innovation enables the lubricating layer to dynamically lock and unlock in response to intense biological fouling in marine environments. The coating effectively addresses the persistent issue of lubricant loss observed in previous SLIPS-based systems, significantly extending its service life in practical marine applications. This breakthrough demonstrates considerable potential for real-world use, offering a promising solution for long-lasting marine antifouling coatings.

#### 4.3.2. Physical Deposition Strategy

The physical deposition strategy offers a versatile and scalable approach to modifying material surfaces by directly applying functional layers, enabling precise control over surface properties for enhanced antifouling performance. Nataraj et al. have developed an innovative coating process for polysulfone membranes using a physical deposition strategy (Figure 7C) [98]. In this process, the polysulfone membrane is sequentially immersed in chitosan and silver nitrate solutions. During this treatment, chitosan acts as a reducing agent, converting silver ions into silver nanoparticles that firmly adhere to the chitosan-coated surface. Unlike the naturally rough and porous structure of polysulfone materials, the incorporation of chitosan-silver nanoparticles results in a significantly smoother surface. This enhanced smoothness markedly improves the membrane’s antifouling performance. By effectively addressing the persistent issue of pollutant adhesion on polysulfone nanofiltration membranes, this method not only increases water flux but also enhances the overall antifouling properties of the interface. This advancement represents a substantial improvement over conventional commercial polysulfone membranes, offering a promising solution for more efficient and durable filtration systems.

#### 4.3.3. Chemical Deposition Strategy

In the human body, natural antifouling mechanisms are exemplified by lubricating coatings, such as those formed by mucins (Figure 7D) [99]. Mucins, a group of glycoproteins with molecular weights reaching up to MDa, are a major component of mucus and serve as excellent lubricants, protecting sensitive cell surfaces from mechanical damage. Inspired by this natural system, Oliver’s research group developed a novel coating process using a chemical deposition strategy [99]. In this process, the methoxy groups on the surface of PDMS samples are first converted to hydroxyl groups. Subsequently, carbodiimide-modified gastric mucin is grafted onto the interface using a silane coupling agent. This covalent coupling enhances the mucin surface’s resistance to stripping, ensuring its stability on various curved interfaces. Protein-based anti-bio-fouling coatings, known for their excellent biosafety, hold significant promise in medical applications. By leveraging the lubricating properties of mucins, these coatings not only prevent fouling but also provide protective benefits to the interface, demonstrating considerable potential for future use in medical and other fields.

## 5. Application Directions of Antifouling Bio-Coatings

Antifouling bio-coatings play a critical role in preventing biofouling by protecting material surfaces from the attachment of contaminants [100]. Initially widely used in ships and marine facilities, antifouling bio-coatings are now increasingly being applied in medical, energy, and other fields. Beyond their primary function of preventing fouling, these coatings significantly reduce maintenance costs while enhancing the lifespan and performance of materials. As eco-friendly antifouling coating technologies continue to advance, their future application prospects are expanding, offering more effective, sustainable, and cost-efficient solutions to combat biofouling challenges.

### 5.1. Marine Antifouling Bio-Coatings

#### 5.1.1. Anti-Bio-Fouling on Ship Surfaces

**Figure 8:**
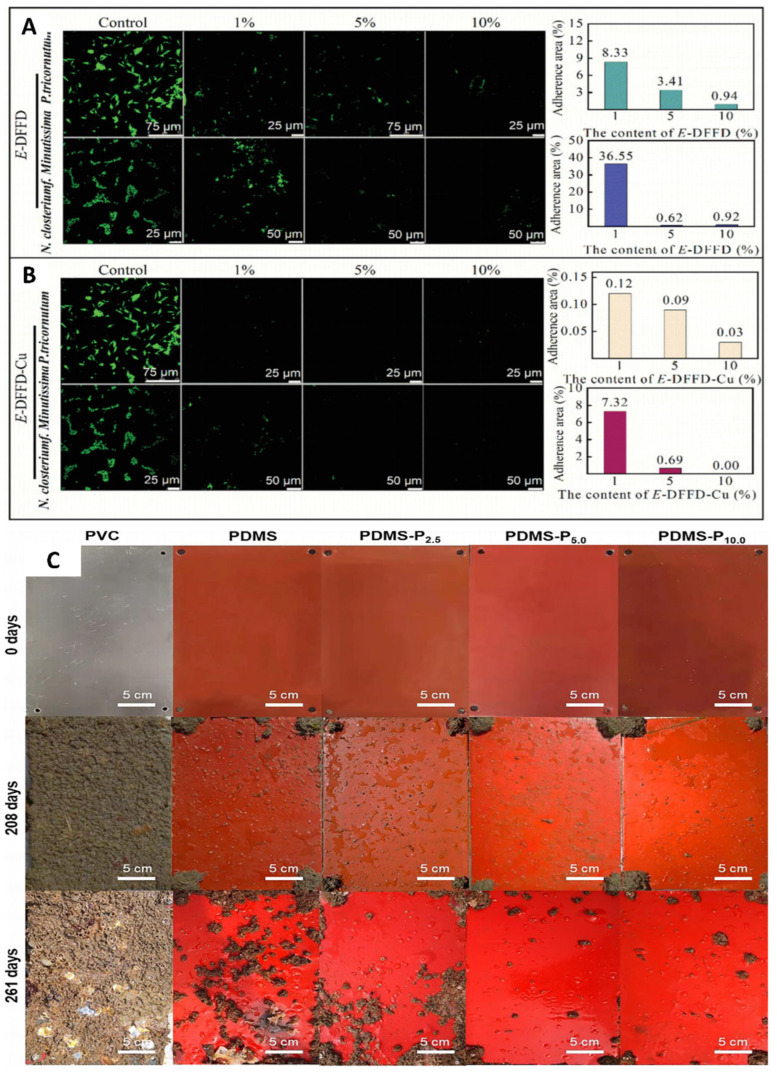
The fluorescence microscopies of P. tricornutum and N. closterium f. minutissima adherence to the coating surface with different contents of (**A**) E-DFFD and (**B**) E-DFFD-Cu. (**C**) Panel data of PDMS coatings and PDMS-Px coatings soaked in the Yellow Sea (36°0839′N, 120°3118′E) for 0, 208, and 261 days. Copyright 2022, Elsevier.

During prolonged exposure to seawater, particularly in static marine environments, the colonization of various algae on surfaces significantly accelerates equipment aging [100]. As a result, preventing algal growth is a critical performance metric for marine antifouling coatings. To address this issue, Zhang et al. developed a bio-based coating that demonstrates effective antialgae and antibacterial adhesion properties [101]. This coating is formed by the complexation of 2,5-diaminofurandione with copper ions, which significantly reduces the minimum inhibitory concentration of copper ions for killing bacteria. In the antialgae experiment, the coating reduces the adhesion area of algae and has an excellent antialgae effect. Meanwhile, the reduction in copper ion concentration reduces the damage of the coating to marine organisms, making it an environmentally friendly antifouling coating. (Figure 8A,B).

The fouling caused by marine organisms such as barnacles and mussels poses significant challenges to the maritime transportation industry, leading to increased operational costs and maintenance burdens [102,103]. To address this issue, Zhang’s research group developed a multifunctional marine anti-bio-fouling coating using a one-pot method [104]. This coating involves the covalent modification of zwitterionic lipids and capsaicin polymers onto the surface of polydimethylsiloxane (PDMS). By combining the protein-repelling properties of zwitterionic pairs with the bactericidal effects of capsaicin, which is continuously released, the coating effectively reduces the adhesion of proteins, bacteria, and other marine organisms. Marine tests demonstrated the coating’s excellent antifouling performance, offering an environmentally friendly and efficient solution for combating marine biofouling (Figure 8C). Although capsaicin has been regarded as an environmentally friendly and nontoxic drug, the characterization of its biocompatibility in this method is lacking.

**Figure 9:**
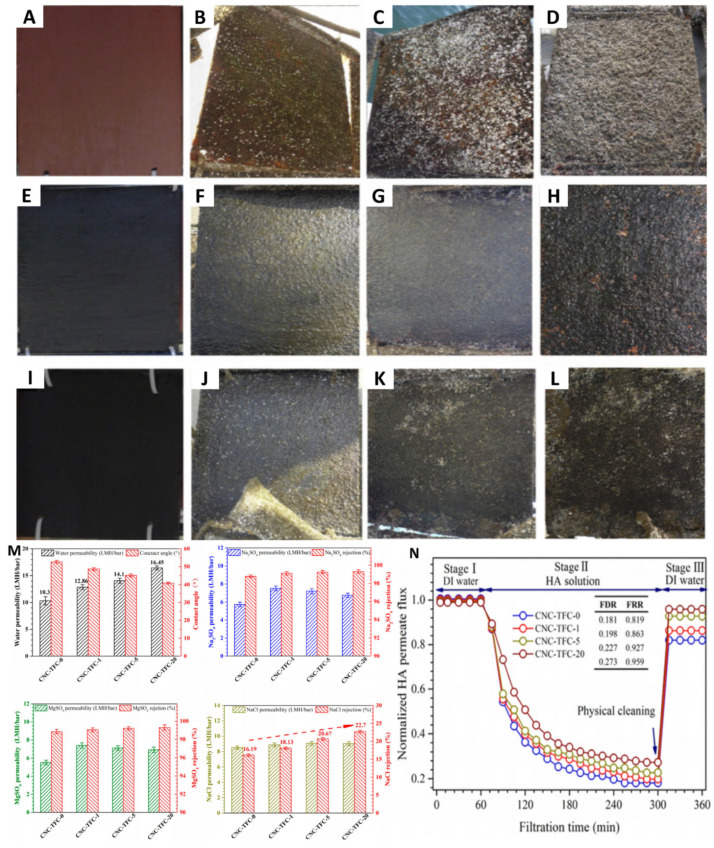
Marine antifouling effect of coatings coated with protease antifouling paint, from left to right are sample photos soaked in the Mediterranean Sea for 0 months, 2 months, 4 months, and 7 months: blank (**A**–**D**), PAP1 (**E**–**H**), and PAP2 (**I**–**L**). Copyright 2015, Elsevier. (**M**) Contact angle of desalination membrane, permeability, and retention rate to sodium sulfate, magnesium sulfate, and sodium chloride. (**N**) Changes in membrane flux during scaling experiments. Copyright 2018, American Chemical Society.

Sea mussels adhere to wet surfaces through intricate biochemical processes, resulting in billions of dollars in annual losses for the marine transportation industry. Central to this adhesion are mussel adhesive proteins, which play a critical role by removing interfacial water and facilitating strong surface bonding [35,105,106]. To combat this issue, Carlos’s research team developed an innovative antifouling coating that specifically targets the protein adhesion step in the mussel attachment process. This coating incorporates proteases, which hydrolyze adhesive proteins, encapsulated within rosin. The solution is dissolved in methyl ethyl ketone and applied to surfaces, effectively preventing protein adhesion and enhancing water resistance [107]. Papain, a key protease in the coating, interacts with and disrupts mussel adhesion upon contact. Extended marine experiments have shown that surfaces treated with this antifouling coating remain significantly cleaner over prolonged periods in oceanic conditions (Figure 9A–L), confirming the exceptional antifouling performance of the papain-based coating. Although this method mentions that the replacement of metal ions by proteases can mitigate the negative impacts on the environment, there is no actual data to support this claim.

#### 5.1.2. Seawater Desalination

Seawater desalination, the process of removing salt from seawater to produce fresh water, offers a safe, reliable, and climate-resilient water source. The three primary desalination technologies are membrane-based, thermal, and emerging processes [108,109]. Among these, nanofiltration membrane-based technology stands out due to its superior filtration efficiency, high selectivity, and ability to address complex contamination challenges. However, its widespread application is hindered by challenges such as membrane fouling and the need for high throughput [110]. To address these issues, Wiesner’s group developed a bio-based composite membrane designed to enhance both separation performance and antifouling properties. This membrane was created by coupling cellulose nanocrystals (CNC) with piperazine and polymerizing them at the interface to form a CNC-TFC hybrid film [111]. The surface modification of CNC significantly increases the negative charge at the interface, improving the repulsion of like-charged ions (Figure 9M). Additionally, the formation of numerous hydrogen bonds within the membrane enhances water flux and fouling resistance (Figure 9N). The integration of high throughput, superior separation efficiency, and robust antifouling capabilities represents a key advancement in the development of next-generation seawater separation membranes.

### 5.2. Medical Antifouling Bio-Coatings

#### 5.2.1. Antifouling of Implant Surfaces

Bio-inert materials capable of resisting the adsorption, adhesion, and activation of plasma proteins, platelets, red blood cells, and white blood cells are essential in biomedical applications, particularly for antithrombotic implant devices and hemodialysis membranes [112,113,114,115,116,117]. Unlike marine antifouling materials, blood-contacting antifouling materials must not only achieve effective antifouling performance but also ensure high biosafety due to their demanding usage conditions. This dual requirement represents the primary focus for the development of bio-based antifouling materials in the biomedical field.

**Figure 10:**
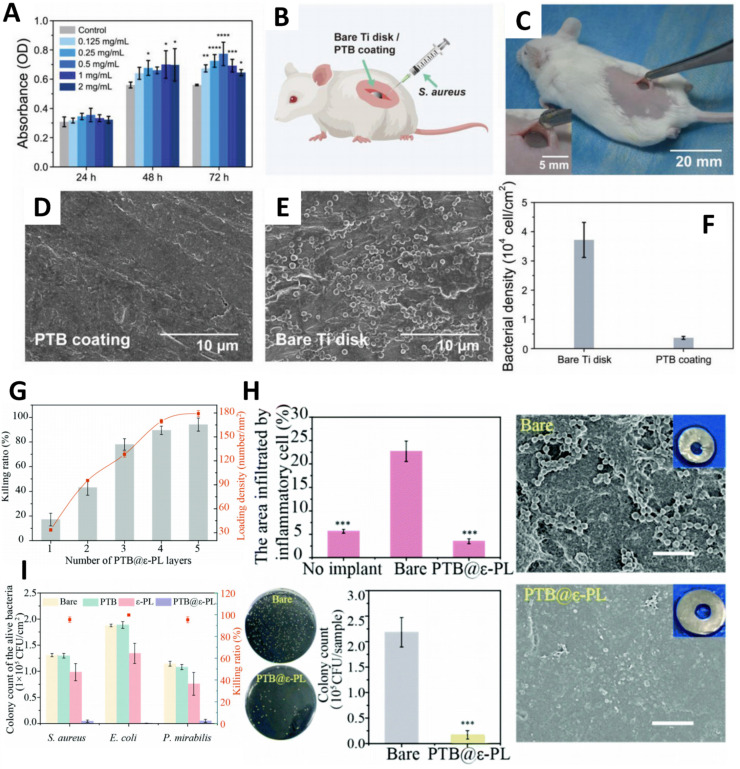
Performance of the membrane: (**A**) Cytotoxicity test of protein membranes. error bars represent means ± SD for n = 5, * *p* < 0.05, ** *p* < 0.01, *** *p* < 0.001, and **** *p* < 0.0001. (**B**,**C**) Animal experimental models with photographs. (**D**,**E**) SEM images of the results of antiadhesion experiments in vivo. (**F**) Histogram of the density of bacteria on the surface. (**G**) The influence of repeated coating times on the antibacterial effect against *S. aureus* and the average loading density of ε-PL immobilized in the PTB@ε-PL nanofilms. (**H**) The number of viable *S. aureus* recovered from sample surfaces in subcutaneous rat model (n = 6, *** *p* < 0.001); optical photos (inset) and SEM images showing Ti with PTB@ε-PL coating and bare Ti surfaces after 3 days of implantation in vivo. Scale bars in SEM are 5 μm. (**I**) The antibacterial effects of PTB, ε-PL adsorption layer on glass, and PTB@ε-PL coating against *S. aureus*, *E. coli*, and *P. putida* (bare glass as control group). Data are shown as mean ± SD. n = 5 per group.

When blood comes into contact with a surface, plasma proteins such as thrombin can adsorb onto the surface, initiating the formation of thrombi. This process can further trigger inflammation and infection [118]. Blood contamination typically begins with the adhesion of platelets to the surface, with fibrinogen adhesion playing a pivotal role in this process. Inhibiting fibrinogen adhesion is therefore a key strategy for effectively reducing blood contamination [119]. Phase transition bovine serum albumin (PTB) film, composed of continuous, dense, colorless, and transparent oligomer particles, offers a promising solution. The film’s thickness is precisely controllable, enabling stable and robust adhesion to various substrates. Even under extreme conditions, the film maintains its original morphology and thickness. Experimental results demonstrate that PTB film exhibits excellent antiadhesion properties against a wide range of contaminants, including proteins, physiological fluids, and extracellular matrices. Moreover, it effectively resists the adhesion of bacteria, fungi, biofilms, cells, and platelets. Importantly, PTB is composed of proteins, which are FDA-approved biopharmaceuticals in the United States. Cytotoxicity and histological analyses of rat subcutaneous implantation have confirmed the film’s outstanding biosafety (Figure 10A), highlighting its potential for biomedical applications (Figure 10B–F).

Implantable biomedical devices have seen remarkable advancements, yet they continue to face significant challenges related to biomedical infections [117]. When bacteria adhere to the surface of these devices, they secrete extracellular matrices that encapsulate the bacteria, forming biofilms that lead to persistent infections. To address this issue, ε-Polylysine (ε-PL), an antimicrobial peptide composed of 25–35 lysine residues and approved by the U.S. Food and Drug Administration (FDA) as a food preservative in 2003, has been utilized. Yang’s research group has successfully immobilized ε-PL onto medical material surfaces using PTB. This innovative approach combines the antifouling properties of protein films with the potent bactericidal activity of antimicrobial peptides (Figure 10G) [120]. Animal bacterial infection experiments further confirmed the coating’s excellent antibacterial performance (Figure 10H,I). These findings highlight PTB@ε-PL as a highly promising multifunctional coating, capable of preventing biomedical-related infections and significantly extending the lifespan of various biomedical implants.

#### 5.2.2. Antifouling of Medical Device Surfaces

Domestic medical devices, designed for detection, treatment, healthcare, and rehabilitation purposes, are increasingly being adapted for home use. However, under the complex and varied conditions of household environments, these devices are prone to bio-fouling, where biological contaminants accumulate on their surfaces. This issue poses a significant barrier to their large-scale commercialization [121,122]. To address this challenge, the development of antifouling coatings that effectively reduce surface contamination represents a viable and promising solution.

**Figure 11:**
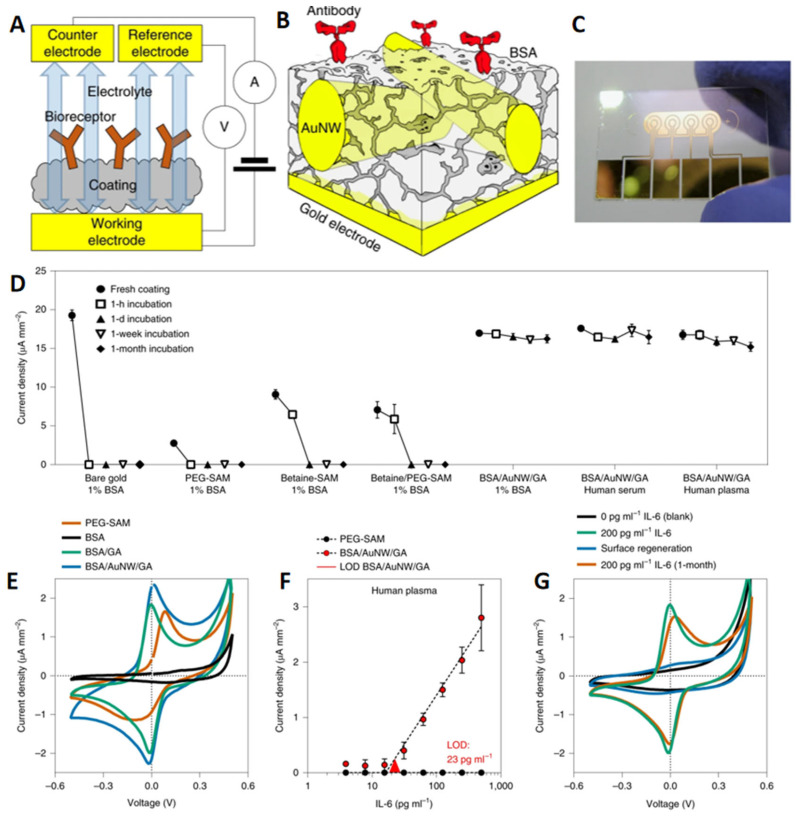
(**A**) Schematic diagram of the three-electrode electrochemical setup used. (**B**) Schematic diagram of an antibody-functionalized BSA/AuNW/GA coating on a gold electrode. (**C**) Photograph of the electrode chip, which consists of four working electrodes with a common reference and counter electrode. (**D**) Comparison of current density averages recorded on bare gold electrodes, PEG-SAM modified electrodes, betaine-SAM-, betaine/PEG-SAM, and optimized BSA/AuNW/GA coated electrodes, stored in 4 °C in 1% BSA, human serum, or human plasma for >30 days (n = 4 independent electrodes, error bars represent standard deviation of the mean). (**E**) Comparison of TMB oxidation and reduction peaks on IL-6 biosensors prepared using BSA/GA, PEG-SAM, and BSA/AuNW/GA (assay performed in PBS). (**F**) Calibration curves for IL-6 detection in unprocessed human plasma on BSA/AuNW/GA coated electrodes (red) and electrodes prepared using PEG-SAM-based surface chemistry (black). n = 4 independent electrodes, circles represent mean values of current density, error bars represent standard deviation of the mean. (**G**) Cyclic voltammograms recorded after completing the IL-6 assay on fresh BSA/AuNW/GA coated electrodes, and after regeneration and repeated assays of captured antibodies in glycine one month later. Copyright 2024, Elsevier.

Donald et al. developed an advanced antifouling coating for domestic electrochemical detection electrodes [123], composed of bovine serum albumin (BSA), gold nanowires (AuNWs), gold nanoparticles, or carbon nanotubes (CNTs), along with other conductive nanomaterials (Figure 11A–C). This coating utilizes BSA to nonspecifically block protein adsorption while enabling the effective diffusion of soluble electroactive substances to the electrode. Remarkably, even after one month of exposure to processed human blood or plasma, the BSA/AuNW/GA coating retained excellent antifouling properties with minimal loss in electrode sensitivity (Figure 11D–G). These results highlight its potential for applications in invasive medical devices, where maintaining long-term performance and biocompatibility is critical.

#### 5.2.3. Anticoagulant and Anti-Inflammatory Coatings for Medical Devices

**Figure 12:**
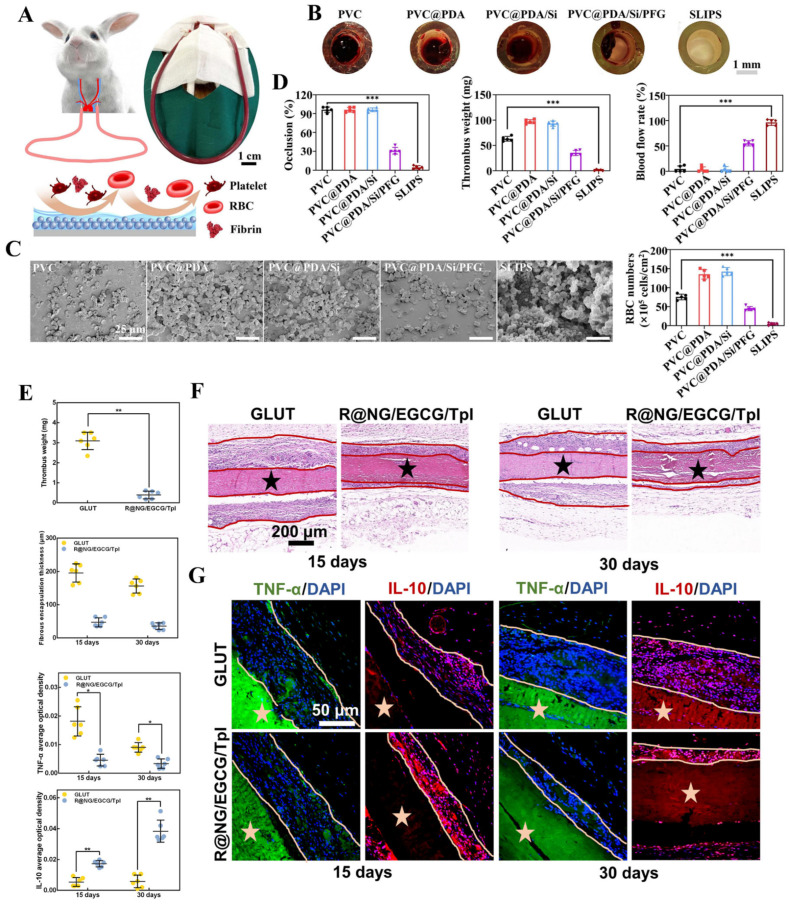
(**A**) Anticoagulant animal models. (**B**) Schematic diagram of antiplatelet and blood cell adhesion on PVC tubes before and after SLIPS modification. (**C**) Scanning electron micrographs of different surface coatings after circulation. (**D**) Results of anticoagulant animal experiments: occlusion rate, thrombus weight, blood flow statistics (*** p < 0.001). Copyright 2022, Elsevier. (**E**) Quantitative data of immunofluorescence analysis of capsules around biological valve leaflets and calcium deposits on biological valve leaflets. Scale bar, 200 μm. * *p* < 0.05, ** *p* < 0.01 (*t*-test, error bars are defined as SD). (**F**) H&E staining photographs after subcutaneous implantation for thirty days. (**G**) Photographs of immunofluorescence analysis of biological valve leaflets. Scale bar, 50 μm.

Blood-contacting medical devices are widely used in clinical treatments, but their performance is often compromised by the complex physiological environment, particularly the tendency of blood to coagulate upon contact with foreign surfaces. This interaction triggers a coagulation cascade, involving protein adsorption, platelet activation, and thrombus formation, leading to significant clinical challenges [124,125]. To address these issues, Huang’s group developed a bio-based antifouling coating designed to minimize blood adhesion on dialysis tubes [126]. The coating is fabricated through the stepwise deposition of polydopamine (PDA), (3-aminopropyl)triethoxysilane (APTES), and fluorinated poly(hexafluorobutyl methacrylate-co-glycidyl methacrylate) (PFG) on the surface, followed by the infusion of lubricating perfluorocarbon oil to create a slippery liquid-infused porous surface (SLIPS). This coating demonstrates excellent biocompatibility and effectively prevents blood adhesion to the device. In vitro experiments revealed that coated instruments exhibited minimal blood adhesion after 30 min of immersion in blood (Figure 12B). Furthermore, in vivo experiments confirmed that the circulatory blood pipeline remained free of coagulation and fully functional throughout the testing period (Figure 12A,C,D). These findings underscore the potential of SLIPS for broader applications in blood dialysis and other medical devices requiring antifouling and antithrombogenic properties.

Coatings for implants, particularly those used in cardiovascular devices, are critical in the treatment of cardiovascular diseases. However, the high risk of thrombosis poses a significant challenge to the effective functioning of these implantable materials. Surface-induced coagulation can trigger a cascade of inflammatory responses, potentially exacerbating the condition and leading to complications such as vascular calcification, degradation, and restenosis. To address these issues, coating modifications have emerged as a widely recognized strategy. Wang et al. developed an innovative coating that integrates both anti-inflammatory and anticoagulant functions [127]. The coating integrates the synergistic anti-inflammatory effects of Tempol, a superoxide dismutase mimetic, and epigallocatechin gallate (EGCG), a polyphenol ester, and the anticoagulant rivaroxaban. These active components are encapsulated within a thrombin-cleavable peptide-linked nanogel. The coating’s effectiveness and multifunctionality were validated through tests on bioprosthetic valves and vascular stents. After 15 days of implantation, hematoxylin and eosin (H&E) staining revealed significantly reduced inflammatory cell infiltration and thinner fibrous membranes, demonstrating a marked decrease in the inflammatory response of the bioprosthetic valves in vivo (Figure 12E–G). The components of the coating are all biopharmaceuticals approved by the US FDA. This advanced coating not only enhances the compatibility of implant interfaces with surrounding tissues but also provides valuable insights for the design of functional endothelial coatings, paving the way for improved outcomes in cardiovascular implant applications.

### 5.3. Antilipid Bio-Coatings

#### 5.3.1. Antilipid Pollution Adhesion

The spreading and adhesion of lipid substances on surfaces are major contributors to oil pollution. Despite their widespread use in daily life and industrial applications, the resource wastage caused by oil adhesion, combined with the excessive use of cleaning agents, significantly escalates carbon emissions. Consequently, the development of oil-resistant interfaces has become a critical priority [128,129]. Heavy components in crude oil, known for their strong surface activity, play a key role in this process. When these components adsorb onto a surface, they alter the interface’s properties, shifting it from hydrophilic to oleophilic, thereby exacerbating oil adhesion and pollution. Addressing this issue through innovative oil-resistant interface technologies is essential for reducing environmental impact and improving resource efficiency.

**Figure 13:**
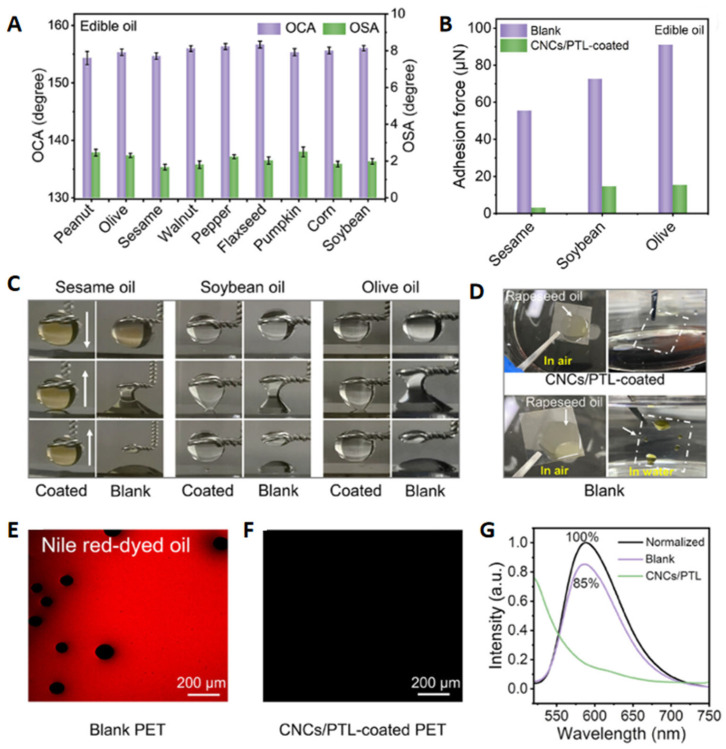
Evaluation of the wettability and adhesion of edible oils on the surface of a cellulose nanocrystal/phase-transition lysozyme (CNC/PTL) coating. (**A**) Underwater oil contact angles (OCAs) and oil sliding angles (OSAs) of various edible oils on the CNC/PTL coated surface. (**B**) Measurement of underwater oil adhesion forces on CNC/PTL coated and blank silicon wafer surfaces. (**C**) Optical images of oil droplet shapes captured during the adhesion measurement process (contact and separation of the oil droplet with the substrate surface). (**D**) Self-cleaning of a glass surface contaminated with rapeseed oil in water. Laser scanning confocal microscopy (LSCM) images showing Nile red oil adherence to (**E**) blank and (**F**) CNC/PTL-coated polyethylene terephthalate (PET) surfaces, with corresponding fluorescence spectra, as shown in (**G**).

Yang’s group developed a phase transition lysozyme (PTL)/cellulose nanocrystal (CNC) composite coating using a layer-by-layer self-assembly method [130]. This coating demonstrates exceptional mechanical stability and regenerative properties, maintaining its robustness in practical applications over an extended lifecycle with simple immersion. Notably, the coating reduces the adhesive force of oil on common substrates by a factor of 20 (Figure 13A,B). Experimental results reveal that oils with varying viscosities and surface tensions exhibit ultra-low adhesion to the CNC/PTL coating in water, with rolling angles consistently below 10° (Figure 13C). In contrast, the same oils display significantly higher underwater oil contact angles and rolling angles above 90° on bare glass surfaces, indicating strong adhesion. High-speed camera analysis further highlights the coating’s excellent self-cleaning properties, while Nile red staining visually confirms its superior underwater oleophobicity (Figure 13D–G). These findings underscore the coating’s potential for applications requiring effective oil resistance and self-cleaning capabilities.

**Figure 14:**
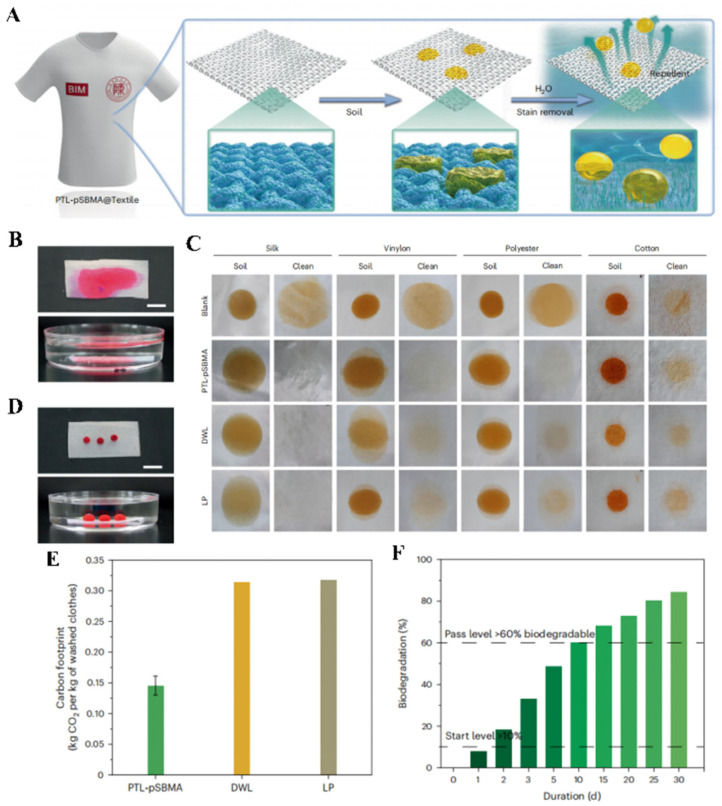
(**A**) Coating is an effective way to solve the contamination of fabric surfaces. (**B**) Photograph of contaminated fabric. (**C**) Optical photos of antifouling experiments before and after coating on various fabric surfaces. (**D**) Optical photos of the underwater oleophobicity of the fabric after coating. (**E**) Complete aerobic decomposition of the PTL-pSBMA dispersion, as measured via the BOD of the microorganisms in the test solution. (**F**) Comparison of water and electricity consumption between PTL-pSBMA and commercial DWL and LP.

To address the issue of cleaning contaminated clothing, substantial amounts of surfactants are utilized in daily life. However, the discharge of these surfactants has led to pollution of land, water bodies, and the overall ecological environment. Constructing antifouling and oil-repellent coatings on fabric surfaces emerges as a novel strategy to tackle this problem (Figure 14A). Yang’s group has employed a protein phase transition strategy, using zwitterion-modified lysozyme, to fabricate an oil-resistant coating on textile surfaces [80]. This coating demonstrates excellent underwater oleophobic properties and effectively reduces oil adhesion on various types of fabrics (Figure 14B–D). Consequently, it significantly decreases the use of surfactants during the cleaning process, offering a new avenue for reducing carbon footprint and promoting a greener lifestyle (Figure 14E,F).

The phenomenon of crude oil adhesion to surfaces arises from the interplay of multiple mechanisms, resulting in a complex interfacial chemical environment that poses significant challenges for achieving effective oil resistance. While current oleophobic coatings show promise, their performance in complex and dynamic real-world environments remains an area requiring further investigation. Continued research is essential to optimize these coatings for practical applications, ensuring they can reliably address oil adhesion under diverse and demanding conditions.

#### 5.3.2. Oil–Water Separation

**Figure 15:**
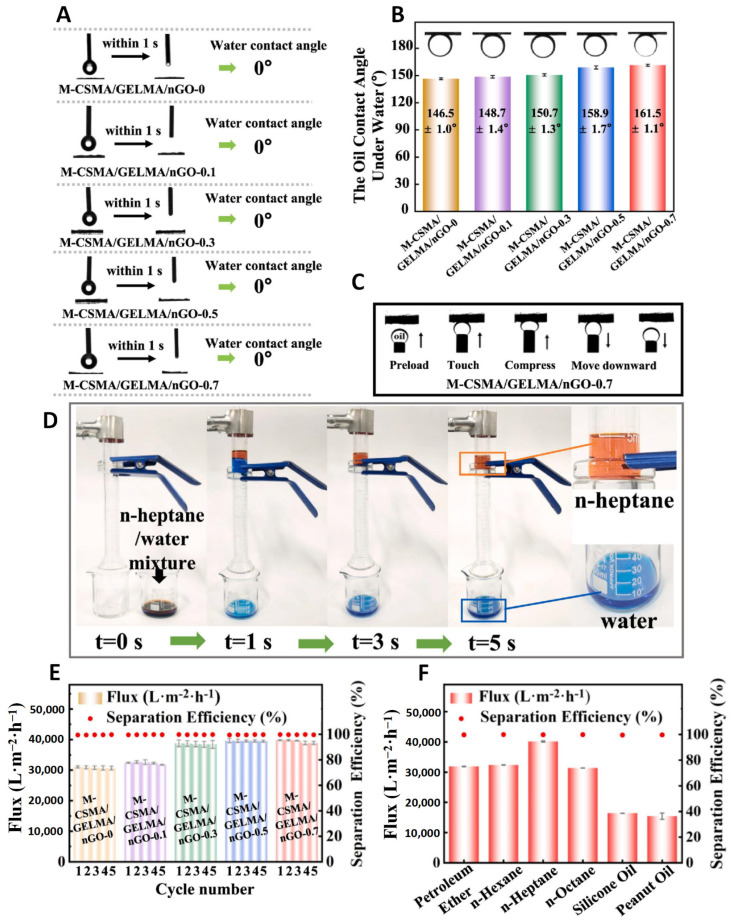
(**A**,**B**) Variation in material water contact angles/underwater oil contact angles with graphene concentration. (**C**) Dynamic underwater oil adhesion test. (**D**) Schematic diagram of the oil–water separation model. (**E**) Separation efficiency and water flux in a five-cycle experiment separating a heptane/water mixture. (**F**) Separation efficiency and water flux for different oil–water mixtures. Copyright 2024, Elsevier.

With the rapid growth of petroleum-related industries, offshore oil spills and the discharge of industrial organic wastewater have become frequent occurrences, posing severe threats to the ecological environment [131,132]. To address this pressing issue, the development of bio-based films with exceptional antifouling properties and oil–water separation capabilities has emerged as a highly effective solution. Wu’s research group successfully fabricated an antifouling oil–water separation membrane using 3D printing technology, employing methacrylated chitosan, gelatin, and graphene as bio-inks (Figure 15A,B) [133]. Dynamic oil fouling adhesion experiments confirmed the membrane’s excellent antifouling performance (Figure 15C). Furthermore, oil–water separation tests demonstrated its high efficiency in separating various oils, including diethyl ether, n-hexane, n-heptane, n-octane, silicone oil, and peanut oil. Notably, the membrane achieved near 100% separation efficiency for the n-heptane/water system (Figure 15D–F) while maintaining a remarkably high water permeation flux. This bio-based membrane, produced through 3D printing, showcases outstanding separation performance and holds significant promise for practical applications in addressing petroleum pollution.

### 5.4. Antiscale and Anti-Ice Bio-Coating

#### 5.4.1. Antiscale Coatings for Metal Surfaces

**Figure 16:**
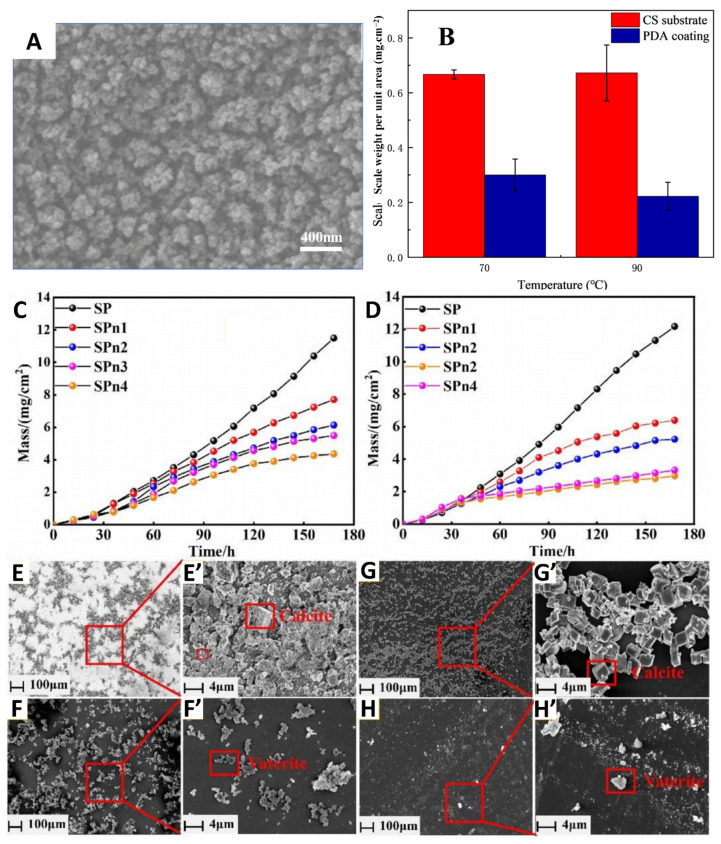
(**A**,**B**) Morphology of dopamine coating by scanning electron microscope and its antifouling effect. (**C**,**D**) Curves showing the mass change in coatings under different pH conditions for scale inhibition. (**E**,**E’**) SEM images of blank substrate under supersaturated calcium carbonate at pH = 10. (**F**,**F’**) SEM images of coating under supersaturated calcium carbonate at pH = 7. (**G**,**G’**) SEM images of coating under supersaturated calcium carbonate at pH = 10. (**H**,**H’**) SEM images of coating under supersaturated calcium carbonate at pH = 7. Copyright 2022, Elsevier.

Antifouling on metal surfaces carries significant economic importance. The deposition of scale, dirt, and other contaminants on metal surfaces gradually reduces the flow channels or gaps in pipes, valves, heat exchangers, and other equipment, ultimately leading to blockages. The presence of a scale layer increases the roughness of the metal surface, thereby enhancing the friction between contact surfaces and accelerating the wear of metal components. To address this issue, Wang’s research group constructed a polydopamine coating on the metal surface through electrodeposition, which only takes three minutes to build an antifouling coating on the metal surface (Figure 16A,B) [134]. The antifouling performance of the coating was evaluated by weight measurement, and the data showed that the coating could reduce the surface scale mass at different temperatures, with better scale inhibition effect at high temperatures.

#### 5.4.2. Antiscale Coatings

The formation of scale in energy transmission processes can significantly reduce efficiency and impair heat transfer, leading to substantial operational losses [135]. Traditional scale removal methods, such as physical cleaning, magnetic fields, and electronic fields, are often hindered by high costs, excessive energy consumption, and environmental concerns [136,137]. To address these challenges, the development of advanced antifouling interface materials has emerged as a promising solution. The Wang research group pioneered a pH-responsive smart coating, by first preparing porous polysulfone (PSF) microcapsules to serve as carriers. The active scale-inhibiting agent, 1-hydroxyethylidene-1,1-diphosphonic acid (HEDP), was encapsulated within these microcapsules, with stearic acid used as the outer coating to create a pH-responsive smart microcapsule system. Experimental results demonstrate that the composite coating exhibits exceptional scale inhibition properties, particularly in alkaline environments, where its performance is further enhanced (Figure 16C–H). [138] This innovative approach highlights the potential of smart coatings to effectively mitigate scale formation while minimizing environmental impact.

#### 5.4.3. Anti-Icing Coatings for Metal Surfaces

**Figure 17:**
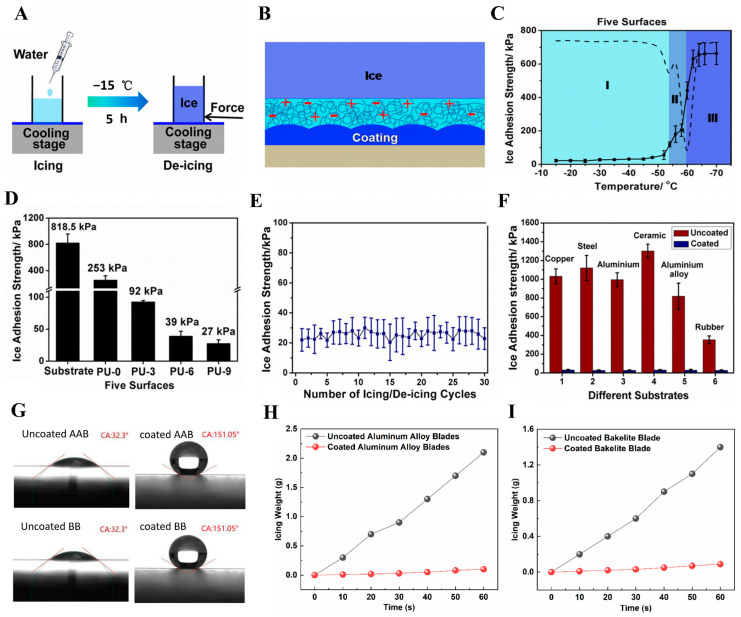
Anti-icing performance testing device and its effects: (**A**) Schematic diagram of the device used for testing ice adhesion strength. (**B**) Mechanism of introducing an aqueous lubrication layer to reduce ice adhesion strength. (**C**) Ice adhesion strength of PU-9 at different temperatures. (**D**) Ice adhesion strength on PU coatings with different hydrophilic component weight contents. (**E**) Change in ice adhesion strength on the PU-9 coating with freeze–thaw cycles. (**F**) Change in ice adhesion strength before and after coating application. Copyright 2015, American Chemical Society. (**G**) Water contact angle before and after surface coating of wind power equipment. (**H**) Anti-icing effect before and after surface coating of Aluminum Alloy Blades. (**I**) Anti-icing effect before and after surface coating of Bakelite Blade.

The formation of ice on various surfaces can lead to significant problems in daily life [139] and, in extreme cases, catastrophic events such as airplane crashes and power grid failures. To address this issue, scientists have focused on developing strategies to delay or prevent ice formation [140,141]. The Wang’s research group made significant progress by creating a lubricating anti-icing coating using a spin-coating strategy [142]. This coating is based on polyurethane as the primary component, with the hydrophilic dihydroxymethylpropionic acid introduced through covalent bonding and chain extension via reaction with isophorone diamine. The hydrophilic and hydrophobic segments self-assemble into nanoparticles in solution, which are then applied to the surface through spin-coating. Compared with uncoated substrates, the modified interface exhibited a 75% reduction in ice adhesion strength (Figure 17A,B). This reduction is attributed to the formation of an aqueous lubrication layer between the ice and the substrate surface (Figure 17C–E). The lubrication layer acts as a surface lubricant, minimizing surface defects and preventing mechanical interlocking between the ice and the substrate. By incorporating hydrophilic polymers to create this lubrication layer, the coating significantly reduces ice adhesion. Its effectiveness has been validated through multiple freeze–thaw cycles and applications on various substrates (Figure 17F), demonstrating its strong potential for practical use in anti-icing applications.

#### 5.4.4. Anti-Icing Coatings for Wind Turbine Facilities

Icing poses a significant challenge to wind turbines, as it can reduce rotational speed due to accumulation on blade surfaces and cause imbalances or catastrophic failures from uneven distribution. Li’s research group has addressed this issue by developing a hydrophobic anti-icing coating composed of polyvinylidene fluoride (PVDF), N-methylpyrrolidone (NMP), and corn stover biochar residue. This coating is applied to the wind turbine blade surface and exhibits a high contact angle of up to 150° (Figure 17G) [143]. Wind tunnel tests demonstrated that it significantly reduces ice accumulation and enhances ice adhesion strength, making ice removal easier (Figure 17H,I). Given the frequent disruption caused by icing, the use of such antifouling coatings represents a promising strategy for both prevention and mitigation.

## 6. Conclusions

In recent years, there has been significant progress in the development of environmentally friendly antifouling bio-coatings aimed at tackling interface pollution (Table 2). This paper reviews the importance of bio-based antifouling coatings, exploring the mechanisms of pollutant adhesion, construction methods, and practical applications. At the mechanistic level, the chemical, physical, and mechanical interactions between pollutants and surfaces are crucial. These interactions change over time and space, affecting how pollutants adhere. The effectiveness of antifouling coatings depends on surface properties such as free energy, wettability, microstructure, and superlubricity. By applying these principles, bio-based coatings with superhydrophilic or superhydrophobic surfaces, as well as superlubricant interfaces, can be created. Such coatings have significant potential in areas like marine antifouling, medical applications, and anti-icing or antiscaling technologies. Despite these advancements, challenges remain. Issues such as long-term durability, stability under various environmental conditions, and cost-effectiveness still need to be addressed. While promising in marine and medical fields, these coatings must overcome barriers to scalability and real-world performance.

Future research on antifouling bio-coatings should focus on three major directions: developing multifunctional synergistic antifouling systems integrating various antifouling mechanisms; enhancing the long-term stability of coatings under complex conditions (temperature variation, salt corrosion, pH fluctuations); and constructing low-cost large-scale manufacturing processes. Specifically, breakthroughs are needed in the resolution of dynamic interaction mechanisms at the molecular scale (such as pollutant-interfacial energy exchange pathways), the design of salt-resistant and antifouling intelligent polymer networks (light/heat/pH-responsive materials), and the innovation of cross-scale manufacturing technologies to reduce the industrialization threshold. To achieve these goals, interdisciplinary collaborative efforts are urgently needed to promote the large-scale application of bio-based coatings in fields such as marine and medical sectors.

## Figures and Tables

**Table 1:** Preparation strategies, structural accuracy, application potential, cost, durability, and application directions of superhydrophilic coatings.

Strategy	Structural Accuracy	Potential for Large-Scale Development	Applicable Scenarios
Polymer blending strategies,	Middle	High	Industrial antifouling coatings, medical devices
Phase transition self-assembly strategies,	High	Middle	Catalytic carrier, precision sensor
Chemical deposition strategies	High	Low	Electronic devices, corrosion-resistant coatings
Spin-coating strategies	Low	Middle	Laboratory research, optical coating

P.S. Evaluate the structural accuracy of the coating based on its flatness: the rougher the coating is, the lower the accuracy will be; evaluate the potential for large-scale application based on the ease of coating preparation: the simpler the coating process is, the higher the potential will be.

**Table 2:** Classification, examples, and antifouling mechanisms of bio-based antifouling coatings, as well as their advantages and disadvantages.

	Types	Example	Antifouling Mechanism	Advantages and Disadvantages
Natural bio-based coating	Polysaccharide	Cellulose coating	superhydrophobic	Advantage: raw materials are readily available; disadvantage: Insufficient stability
	Protein	Amyloid-like coating	Superhydrophilic	Advantages: there are various functionalization methods; disadvantages: the utilization rate of the reaction is poor
	lipid	Zwitterion coating	Superhydrophilic	Advantages: excellent antifouling performance; disadvantages: weak resistance to salt environment
Synthetic bio-based coating	Biodegradable polymers	Polylactic acid	superhydrophobic	Advantages: degradability is beneficial for environmental protection; disadvantages: the antifouling effect is relatively weak
	Bio-inspired synthetic materials	Polydopamine	superhydrophobic	Advantages: simple preparation; disadvantages: limited antifouling effect.
Composite coating	Inorganic-biological hybrid coating	Titanium dioxide/chitosan	superhydrophobic	Advantages: high strength and wear resistance; disadvantages: limited effect

## Data Availability

No new data were created or analyzed in this study.
